# Quantifying antibiotic resistome risks across environmental niches: the L-ARRAP for long-read metagenomic profiling

**DOI:** 10.1093/bib/bbaf535

**Published:** 2025-10-09

**Authors:** Yongxin Li, Yue Gao, Xiaohui Liu, Yujie Mao, Mingchao Wang, Yunyi Qin, Caili Zhang, Qingru Chen, Kang Ning, Zhi Wang, Maozhen Han

**Affiliations:** School of Life Sciences, Anhui Medical University, Hefei 230032, China; Microbial medicinal resources development research team, Anhui Provincial Institute of Translational Medicine; School of Life Sciences, Anhui Medical University, Hefei 230032, China; Microbial medicinal resources development research team, Anhui Provincial Institute of Translational Medicine; Key Laboratory of Marine Environment and Ecology, Ministry of Education and College of Environmental Science and Engineering, Ocean University of China, Qingdao 266100, China; Key Laboratory for Environment and Disaster Monitoring and Evaluation of Hubei, Innovation Academy for Precision Measurement Science and Technology, Chinese Academy of Sciences, Wuhan 430077, China; Qingdao University of Science and Technology, Qingdao 266000, China; Single-Cell Center, CAS Key Laboratory of Biofuels and Shandong Key Laboratory of Energy Genetics, Qingdao Institute of BioEnergy and Bioprocess Technology, Chinese Academy of Sciences, Qingdao 266100, China; School of Life Sciences, Anhui Medical University, Hefei 230032, China; Microbial medicinal resources development research team, Anhui Provincial Institute of Translational Medicine; School of Life Sciences, Anhui Medical University, Hefei 230032, China; Microbial medicinal resources development research team, Anhui Provincial Institute of Translational Medicine; School of Life Sciences, Anhui Medical University, Hefei 230032, China; Microbial medicinal resources development research team, Anhui Provincial Institute of Translational Medicine; Key Laboratory of Molecular Biophysics of the Ministry of Education, Hubei Key Laboratory of Bioinformatics and Molecular-Imaging, Department of Bioinformatics and Systems Biology, College of Life Science and Technology, Huazhong University of Science and Technology, Wuhan, China; Key Laboratory for Environment and Disaster Monitoring and Evaluation of Hubei, Innovation Academy for Precision Measurement Science and Technology, Chinese Academy of Sciences, Wuhan 430077, China; School of Life Sciences, Anhui Medical University, Hefei 230032, China; Microbial medicinal resources development research team, Anhui Provincial Institute of Translational Medicine

**Keywords:** metagenomic, ARGs, long-read based antibiotic resistome risk assessment pipeline, nanopore sequencing, PacBio sequencing

## Abstract

The global dissemination of antibiotic resistance genes (ARGs) represents a critical challenge to One Health. Existing ARG risk assessment tools (e.g. MetaCompare, ARRI) are constrained by short-read sequencing data, limiting their utility for long-read platforms. To address this gap, we developed the Long-read based Antibiotic Resistome Risk Assessment Pipeline (L-ARRAP), which calculates the Long-read based Antibiotic Resistome Risk Index (L-ARRI) to quantify antibiotic resistome risks. Building upon our previous ARRI framework, L-ARRAP leverages long-read sequencing advantages to concurrently identify ARGs, mobile genetic elements, and human bacterial pathogens, integrating their interactions for risk scoring. Our results showed that L-ARRAP was not only able to accurately identify ARGs and evaluate the antibiotic resistance risk scores in samples of hospital wastewater (HWW), Chaohu lake, and human fecal samples, but also significantly distinguish the ARG risk in HWW samples between before and after disinfection groups, demonstrating the performance of L-ARRAP. Furthermore, L-ARRAP scores exhibited strong concordance with those generated by our laboratory-adapted MetaCompare variant (L-MetaCompare), corroborating its methodological reliability. Overall, to our knowledge, L-ARRAP is the first assessment pipeline of antibiotic resistome for long sequencing reads and has a great potential for monitoring the risk of ARGs in various environmental niches.

## Introduction

The World Health Organization (WHO) has identified antibiotic resistance as a major global health threat [1]. The extensive use of antibiotics in human medicine, agriculture, and animal husbandry has led to the pollution of the environment, thereby promoting the proliferation of antibiotic-resistant bacteria (ARB) and increasing the levels of antibiotic resistance [2–5]. In addition, human bacterial pathogens (HBPs) can recruit antibiotic resistance genes (ARGs) from microorganisms in environments such as soil by horizontal gene transfer (HGT), increasing the potential threat to human health [6–9]. The new concept of ``One Health'' has recently been promoted to simultaneously investigate the effects of antibiotic resistance on humans, animals and environments, and the role of the environment as an important reservoir and transmission channel for ARGs makes the assessment of antibiotic resistance risk (ARR) in different habitats particularly important [5, 10].

Metagenomic sequencing has been widely applied in environmental ARGs research, but short sequencing reads need be assembled for ARGs and genomic background analysis, which may produce incorrect contigs and chimeric assemblies [11]. Moreover, the inherent repeatability of flanking regions of ARGs obtained by HGT would leave many gaps in the assembled genome, leading to fragmented assembly, which makes it challenging to track the source of ARGs [3]. Long-reads produced by Nanopore and PacBio platforms can avoid these limitations by using nonassembly methods to analyze ARGs and its adjacent genes and host.

Current quantitative assessment methods for ARG risk based on next-generation sequencing, such as MetaCompare [12] and ARRI [5], require the counts or abundances of ARGs, mobile genetic elements (MGEs), and contigs of HBPs. However, the contigs from long-reads are too long to accurately reflect the abundances of ARGs and MGEs [13]. The methods from X. Shuai *et al.* [14] and Zhang *et al.* [15] necessitate binning after assembly to calculate the metagenome-assembled genomes (MAGs) of pathogens, but the sequencing data depth of Nanopore and PacBio sequencing is shallower than that of Illumina, making it hard to recover MAGs of low-abundance species and missing key pathogen fragments [16]. Therefore, the methods to investigate ARR based on next-generation sequencing aren't suited to long-reads. Although there are several tools (such as ARGpore2 [3], NanoARG [17], and NanoOK RT [18] for identifying ARGs and their genetic backgrounds in Nanopore metagenomic datasets, there is an absence of a direct quantitative evaluation tool for assessing ARG risks in Nanopore or PacBio metagenomic datasets.

Based on our previously proposed ARRI framework for next-generation sequencing [5], we designed the Long-read based Antibiotic Resistome Risk Assessment Pipeline (L-ARRAP) in this present study. This pipeline not only identifies ARGs, MGEs, and HBPs from Nanopore or PacBio metagenomic data across environmental niches, but also quantifies ARG risk through the Long-read based Antibiotic Resistome Risk Index (L-ARRI), which integrates ARG abundance, mobility potential, and pathogenic host associations. This study first used the L-ARRAP to analyze hospital wastewater (HWW) samples before and after disinfection, finding a significantly higher ARG risk in postdisinfection samples. Next, we analyzed Nanopore-based metagenomic datasets from Chaohu lake and human feces samples to confirm the ability of L-ARRAP and assess ARG risks in different niches. Moreover, the significant positive correlation between L-ARRI and the results of the MetaCompare's modified method (L-MetaCompare) further reinforced the accuracy of L-ARRAP in assessing ARG risks.

## Materials and methods

### Metagenomic data collection and generation

Three representative niches, namely, HWW, human feces, and Chaohu lake, were selected to validate the universality of L-ARRAP. The metagenomic data of HWW and human feces were downloaded from SRA database. Specifically, eight Nanopore samples from HWW (BioProject: PRJNA999488), including four before disinfection samples (M2.1, M2.2, M2.3, and M2.4), and four after disinfection samples (M3.1, M3.2, M3.3, and M3.4) [13]. The metagenomic datasets of 113 human stool samples (BioProject: PRJNA929328) generated by Nanopore sequencing platform were collected [19]. In order to minimize the errors caused by too large or too small sample sizes when calculating abundance, as well as the inter-individual differences caused by data volume, we filtered 26 samples smaller than 100 M and three samples larger than 20 Gb, and downloaded the remaining 84 samples. In addition, four water samples were collected by us in July 2022 from Chaohu lake (Fig. S1 available online at http://bib.oxfordjournals.org/) and sequenced on Nanopore platform. The detailed procedure for sequencing and the information of metagenome data are shown in Supplementary materials and Supplementary Table S1.

### Overview of the L-ARRAP workflow

**Figure 1:**
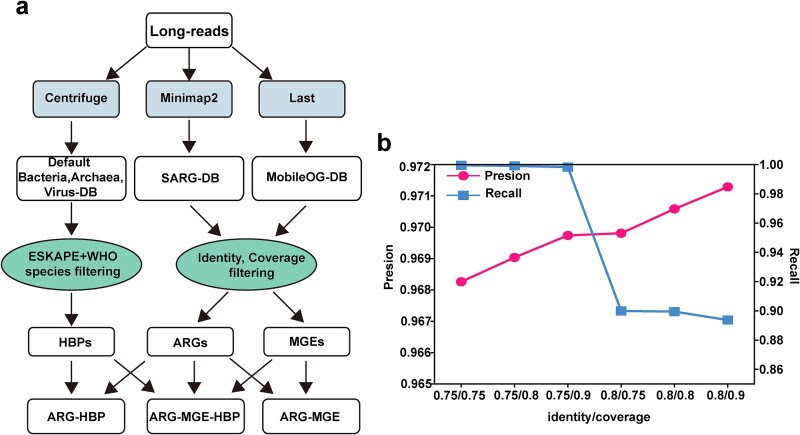
The workflow of L-ARRAP and the impact of different cutoff values on its performance in identifying ARGs. (a) The workflow of L-ARRAP. (b) The precision and the recall of ARGs identified with the different identity/coverage cut-off values by L-ARRAP.

In our study, L-ARRAP was designed for metagenomic data generated from PacBio or Nanopore platforms and can be used to assess the antibiotic resistome risk in different environmental niches. First, Chopper (V8.0.1) [20] was used with ``-q 10 -l 500'' for quality control of reads longer than 500 bp. Second, the Minimap2 (v2.26) [21] and LAST (v2.27.1, −m 100 -D1e9 -K 1) [22] tools were used to align reads to the SARG (v2) [23] and MobileOG-db (Version: Beatrix 1.6 v1) [24] databases, respectively, and the identity >75% and coverage >90% were set to screen out the potential ARGs and MGEs. Third, the taxonomy of reads was annotated by Centrifuge (v1.0.4) [25] and the reads belonging to HBPs were identified by alignment with the HBPs database, which is composed of pathogens from WHO (proposed in 2016) [26] and ESKAPE (proposed in 2024) databases. Fourth, L-ARRAP calculated the abundance of ARG, MGE, and HBP in the sample using the formulas (shown below), respectively. Finally, ARG risk index (L-ARRI) was calculated for each sample. The workflow and detailed methods were illustrated in Fig. 1a and explained in detail below.

### Antibiotic resistance genes and mobile genetic elements identification

To identify ARGs, the best cutoff values of identity/coverage were optimized. A combined dataset of 125 190 non-ARGs bacterial genes was randomly downloaded from the KEGG database [27] and 14 210 ARGs genes from the SARG database were selected and constructed to verify the ARGs annotation precision of L-ARRAP for different cutoff values. Only hits with identical subtype were counted as valid ARGs identification. As shown in Fig. 1b and Fig. S2, the identity cut-off of 75% and coverage cut-off of 90% were selected for ARGs identification (Supplementary Materials). Thus, L-ARRAP calls the Minimap2 (preset map-ont, map-pb, or map-hifi for different reads) to align the reads against the nucleotide sequences of the SARG database (v2.0).

Due to the fact that MGEs are able to capture ARGs from chromosomes and transfer them horizontally to other bacteria via plasmids or phages [28], the roles and abundances of MGEs should be considered when assessing the risk of antibiotics. In this study, MGEs were identified using LAST tool (v2.27.1) to align the reads with the protein database of mobileOG-db (Version: Beatrix 1.6 v1) and then to determine whether they could influence the transfer of ARGs. The MGEs identified by MobileOG-DB can be divided into five groups, including (i) integration and excision (IE) from one genetic locus to another; (ii) replication, recombination, or nucleic acid repair (RRR); (iii) interorganism transfer (T); (iv) element stability, transfer, or defense (STD); and (v) phage (P) specific biological processes (e.g. genome packaging, or lysis and lysogeny). The identity cut-off of 75% and coverage cut-off of 90% are adopted by L-ARRAP, which is similar to ARGpore2's approach [3]. Only the ARG/MGE hits of the highest mapping quality were kept whenever hits overlapped with other ARG/MGE hits by 80% in length to avoid multiple hits on the same gene segment [29].

### Human bacterial pathogens identification

A list of pathogens with critical, high, and medium three priority tiers that are of particular concern with respect to the spread of antimicrobial resistance has been claimed by WHO. Besides, the ESKAPE database [30] harbors multidrug-resistant pathogens that are critical to human health. These two databases were used for the identification of HBPs in L-ARRAP (Table S2 available online at http://bib.oxfordjournals.org/). In this study, the L-ARRAP identifies the reads from HBPs by comparing the TaxID of long sequencing reads annotated with Centrifuge (v1.0.4) with the taxonomic identifiers of pathogens from aforementioned two databases.

### Antibiotic resistome risk evaluation

The reads carried ARGs, MGEs, and belonging to HBPs have been detected, respectively, and then the abundances of components required for risk assessment can be calculated. First, according to the calculation method described by Chen Y *et al.* [31], we calculated the abundances of ARGs and MGEs in following formula:


$$ {\mathrm{Abundance}}_{\mathrm{ARG}/\mathrm{MGE}}\left(\mathrm{coverage},\times /\mathrm{Gb}\right)=\frac{S}{L\times B} $$

Where *S* represents the total coverage of ARG or MGE in the dataset, *L* represents the length of each ARG or MGE, *B* represents the total length of reads with a match in Gb. Namely, the copy number of ARGs and MGEs was calculated as the sum of the alignment coverage over its reference gene for all reads with a match and then normalized to the size of the dataset (Gb) to obtain the abundance of each ARG and MGE subtype. Second, the abundance of ARGs in reads containing both ARG and MGE [Abundance(ARG-MGE)], the abundance of ARGs in reads belonging to HBPs [Abundance(ARG-HBP)], and the abundance of ARGs in reads where both MGE and ARG coexist and belonging to HBP [Abundance(ARG-MGE-HBP)] were calculated with the same calculation formula above. Third, the abundance for each pathogen belonging HBPs was calculated as follows:


$$ {Abundance}_{HBP}\left( coverage,\times / Gb\right)=\frac{R}{G\times B} $$

Where *R* is the total number of base pairs of the reads belonging to HBPs. *G* is the number of base pairs of pathogenic genome to which reads belong. Finally, L-ARRI, which indicates the risk of ARGs, is calculated by L-ARRAP based on the abundances of ARG-MGE, ARG-HBP, and ARG-MGE-HBP using the following equation:


\begin{align*} &\mathrm{L}-\mathrm{ARRI}\\&=\frac{A\left( ARG- MGE\right)+A\left( ARG- HBP\right)+A\left( ARG- MGE- HBP\right)}{A\left( ARG s\right)+A(MGEs)+A(HBPs)}\times{10}^4 \end{align*}

Where *A* is the total abundance sum. The more detailed expressions of all the formulas are in supplementary materials.

### Web service

A web server (http://ncrd.single-cell.cn/index/) was constructed to provide a user-friendly graphical interface for using L-ARRAP to assess the potential ARGs risk. In the analysis section, users can select the cut-off values of Minimap2 and LAST to screen potential ARGs and MGEs. The default Minimap2 and LAST output cutoff values were identity ≥75% and coverage ≥90%, and identity ≥70%, and coverage ≥70%, respectively (Fig. S2).

### Statistical analyses and data visualization

The statistical analyses and visualization were mainly conducted on R platform and R packages, including ``dplyr (v1.1.4)'', ``vegan (v2.6.10)'', ``ggtrendline (v)'', ``ggplot2 (v3.5.1)'', ``venn (v1.11)'', and ``pheatmap (v1.0.12)''.

## Results

### The application of L-ARRAP

To demonstrate the capability of L-ARRAP in profiling ARGs, MGEs, and HBPs and its effectiveness in calculating resistome risk, four predisinfection samples and four postdisinfection samples from HWW were analyzed by L-ARRAP. Then, four samples (CH1, CH2, CH3, and CH4) from Chaohu lake and 84 samples from human feces were analyzed by L-ARRAP to verify its capability in different environmental niches.

#### Dynamic changes of ARGs in HWW samples of before and after disinfection

**Figure 2:**
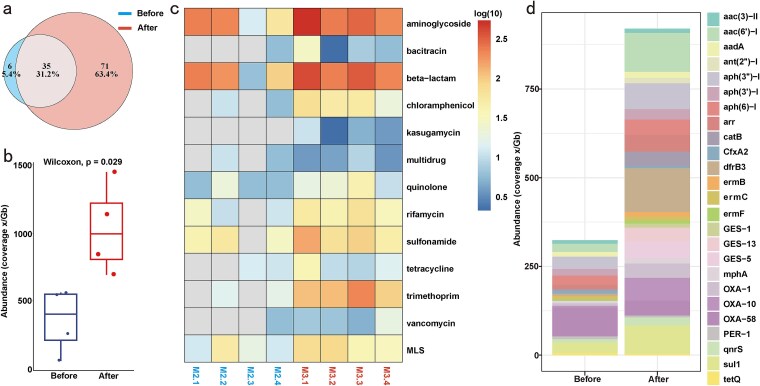
Dynamic of ARGs in the HWW samples of before and after disinfection. (a) Venn plot showed the shared and specific ARG subtypes in before and after disinfection samples. (b) The total abundance of ARGs in after disinfection samples was significantly higher than before disinfection samples. (c) Composition and abundances of different ARG types before and after disinfection. (d) The distribution of top 20 ARG subtypes in HWW samples.

Our results showed that 12 ARGs and 41 ARG subtypes were detected in HWW samples of before disinfection group while 13 ARGs and 106 ARG subtypes were identified after disinfection group (Fig. 2a). In comparison, Rolbiecki D *et al.* [13] reported higher ARG abundance using NanoARG web service compared to L-ARRAP. It may be due to the different databases, identification methods, and cut-off value of annotation parameters used by NanoARG and L-ARRAP, but either method should not affect the results of ARG changes for before and after disinfection groups, and this is indeed the case. The abundance of ARGs in the before disinfection samples was 353.73 ×/Gb (53.05–544.33 ×/Gb), which was significantly lower than after disinfection 1034.43 ×/Gb (691.87–1454.24 ×/Gb, Wilcoxon test, *P* = 0.029, Fig. 2b), and the changes in the abundance of the specific ARGs were shown in Fig. 2c.

Specifically, the ARG subtypes with the highest abundances in before disinfection samples were *OXA-58* (23.0%), *aph(3″)-I* (9.7%) and *sul1* (8.4%). After disinfection, the ARG subtypes with the highest abundances were *dfrB3* (12.0%), *aac(6′)-I* (10.6%), and *sul1* (7.8%, Fig. 2d). Among them, *sul1* was present with high abundance in the samples before and after disinfection. To further verify the accuracy of ARG identification by L-ARRAP, we used LAST software (v2.27.1) to identify ARGs in samples with a cut-off value of 75% similarity and 90% coverage, which was consistent with the method of ARGpore2 for ARG identification. The results obtained were highly similar to those of L-ARRAP. The most abundant ARG subtypes before disinfection were *OXA-58* (20.2%), *aph(3″)-I* (10.5%) and *sul1* (6.3%), and the most abundant ARG subtypes after disinfection were *dfrB3* (13.0%), *aac (6′)-I* (10.0%), and *sul1* (6.4%). What's more, the related studies have shown that *dfrB3* is significantly higher in freshwater than in wastewater microbiomes [32] and *sul1* is one of the most prevalent ARGs detected in waste water treatment plant [33, 34]. Therefore, these results indicated that L-ARRAP could effectively identify ARGs in HWW samples.

#### Distribution of MGEs and their associated ARGs in HWW samples

Numerous studies have shown that the widespread of ARGs is greatly driven by MGEs [28, 35, 36]. In addition, the combination of multiple resistance genes coexisting with mobile elements or on mobile plasmids may cause greater environmental or health risks [5]. Therefore, assessing the distribution of ARGs and MGEs is essential when evaluating ARG risk.

**Figure 3:**
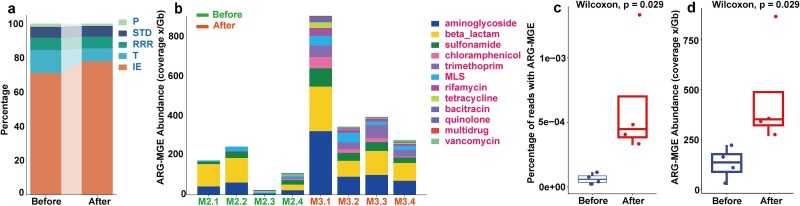
Distribution of MGEs and mobility of ARGs in samples of before and after disinfection groups. (a) Distribution of MGEs concurrent with ARGs. (b) The abundance of ARGs that co-occurring with MGEs. The abundance of ARG-MGE (c) and the proportion of reads (d) with both ARG and MGE in the samples of after disinfection were significantly higher than those in before disinfection group, indicating that the mobility of ARG in the samples after disinfection was higher than that before disinfection.

The result of MGEs co-occurring with ARGs identified by L-ARRAP was shown in Fig. 3a, which revealed that integration and excision (IE, 66.2%–89.3%) was the most frequently identified MGEs in the proximity of ARG identified by L-ARRAP while phage-mediated biological processes (phage, P) were least frequently identified. The results of ARG analysis of HWW samples co-presented with MGE by L-ARRAP in before and after disinfection groups showed that eight ARGs and 29 ARG subtypes were found in the samples of before disinfection group, and 12 ARGs and 87 ARG subtypes were found in after disinfection group (Fig. 3b). The abundance of ARG fractions identified on MGEs increased from 127.8 ×/Gb on average (21.18–221.32 ×/Gb) in before disinfection samples to 457.58 ×/Gb on average (266.16–863.69 ×/Gb) in after disinfection samples (Wilcoxon test, *P* = 0.029, Fig. 3c). The proportion of reads containing both ARGs and MGEs increased from 0.0063% (0.0012%–0.012%) in before disinfection samples to 0.064% (0.032%–0.13%) in after disinfection samples (Wilcoxon test, *P =* 0.029, Fig. 3d). The increase in ARGs abundance and mobility potential after disinfection was in agreement with the findings of Rolbiecki D *et al.* [13]. The above results demonstrate that L-ARRAP is effective and accurate in the identification of MGEs, and the evaluation of ARG mobility.

#### Critical bacterial pathogens in HWW samples

**Figure 4:**
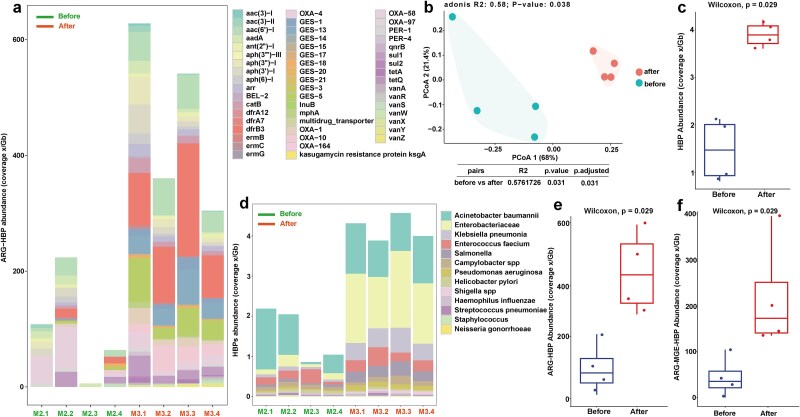
Changes of HBPs and the ARR in before and after disinfection samples. (a) The relative abundances of ARG subtype in the genomes of HBPs. (b) The results of PCoA revealed a significant difference in the distribution of HBPs in the samples before and after disinfection. (c) The abundances of HBPs in samples of before and after disinfection group. (d) Composition of HBPs in samples of before and after disinfection group. The abundances of ARG-HBP (e) and ARG-MGE-HBP (f) in the samples of after disinfection group were significantly higher than those in before disinfection group.

**Figure 5:**
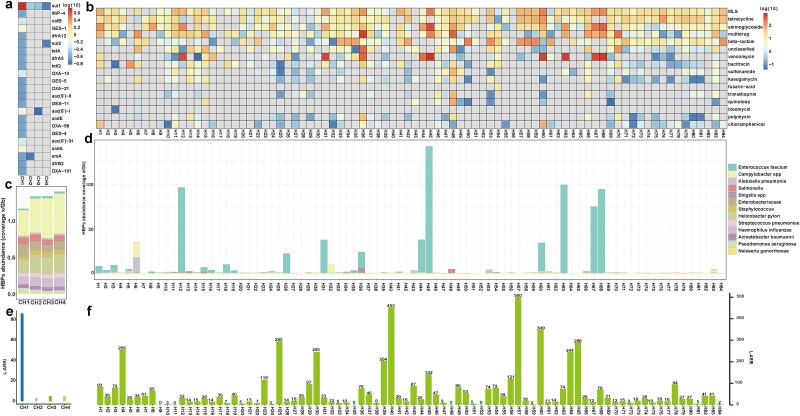
Analysis results of L-ARRAP on components associated with ARR in samples of Chaohu lake and human gut microbiome. (a) The abundances of ARG subtypes (log10) in Chaohu lake. (b) The abundances of ARG types in human gut microbiome. The composition of HBPs annotation in Chaohu lake (c) and human gut microbiome (d). The risk scores of Chaohu lake (e) and gut microbiome (f).

L-ARRAP results showed that a total of 13 pathogens with 19 ARG subtypes were detected before disinfection group, while the same 13 pathogens with 53 ARG subtypes were detected after disinfection group (Fig. 4a and Fig. 4f). Principal coordinate analysis (PCoA) showed that the structure of HBPs was also different before and after disinfection (Fig. 4b). The abundance of HBPs increased from 1.63 ×/Gb before disinfection to 4.19 ×/Gb after disinfection (Wilcoxon test, *P* = 0.029, Fig. 5c). Specifically, the HBP with the highest abundance in before disinfection was *Acinetobacter baumannii* (0.758 ×/Gb before disinfection; 1.07 ×/Gb after disinfection), while the highest abundance in after disinfection was *Enterobacteriaceae* (0.16 ×/Gb before disinfection; 1.61 ×/Gb after disinfection, Fig. 4d). It was worth noting that *A. baumannii*, a critical pathogen in WHO, had a high abundance in the samples of before and after disinfection (0.76 ×/Gb before disinfection, accounting for 44.1% of the total abundance, and 1.07 ×/Gb after disinfection, accounting for 27.5% of the total abundance), which indicated that *A. baumannii* should be paid special attention to in these HWW samples.

Results from L-ARRAP revealed that disinfection significantly increased the abundance of ESKAPE bacteria, WHO priority pathogens, and both ARG-HBP and ARG-MGE-HBP. Specifically, the median abundance of ARG-HBP increased from 97.57 ×/Gb (6.06–218.67 ×/Gb) before disinfection to 442.18 ×/Gb (293.99–609.31 ×/Gb) afterward (Wilcoxon test, *P* = .029; Fig. 4e). Similarly, the median abundance of ARG-MGE-HBP increased from 43.63 ×/Gb (0–174.54 ×/Gb) to 219.50 ×/Gb (135.3–878.0 ×/Gb) (Wilcoxon test, *P* = .029; Fig. 4f). The increase in the abundance of HBPs, ARG-HBP, and ARG-MGE-HBP revealed by L-ARRAP is consistent with the findings of Rolbiecki D *et al.*, who reported a higher abundance of pathogenic taxa and priority resistance determinants in HWW after disinfection [13].

#### The application of L-ARRAP in different environmental niches

Four Nanopore datasets from Chaohu lake and 84 Nanopore datasets from human gut microbiome were used to further verify the risk assessment accuracy of L-ARRAP in different environmental niches. The results from L-ARRAP showed that seven ARG types and 23 ARG subtypes with the total abundance of 11.13 ×/Gb were detected in CH1, whereas only one to two ARG subtypes were identified in the remaining three Chaohu samples. However, our previous study on Chaohu lake based on next-generation sequencing showed that samples from all locations were enriched with ARGs [5]. To further identify ARG in Chaohu lake, ARGpore2 and LAST tools were used to detect ARG in samples from Chaohu lake, but the final results were the same as L-ARRAP, and no more ARGs were found. Despite this, *sul1* was detected in all samples and was the most abundant ARG subtype in CH1 samples, but it was not found in the samples of human gut microbiome (Fig. 5a). Previous studies have demonstrated that *sul1* is one of the most frequently detected ARGs in rivers and lakes [37–39].

Results of the 84 human gut microbiome samples showed that a total of 16 types of ARG and 165 subtypes were identified. The most abundant ARG type in all samples was vancomycin, followed by multidrug, aminoglycoside and macrolide-lincosamide-streptogramin (MLS). Notably, vancomycin was found in only 32 samples, but was abundant in nine samples, resulting in the highest abundance (Fig. 5b). Interestingly, the results of L-ARRAP's ARG prediction for 84 feces samples were similar to those of Shuai M *et al.* that studied drug resistance in gut microbes in healthy populations and type 2 diabetic patients by next-generation sequencing [40].

The four samples from Chaohu lake showed four similar total abundances and species compositions of HBPs (1.09–1.34 ×/Gb, Fig. 5c), with *Campylobacter* as the main causative agent (32.0% ~ 42.6%). However, contrary to the samples from Chaohu lake and HWW, the composition of HBPs varied significantly among different samples of the gut microbiome. Particularly, the abundance of *Enterococcus faecium* was high in several samples (Fig. 5d). The presence of *E. faecium* in pre-Tx alloHSCT samples in high abundance was also confirmed by the results of Shuai M *et al.* More importantly, it was also the only highly abundant pathogen in the L-ARRAP’s HBPs database identified by Spohr P *et al.* [19]. In addition, the risk scores of Chaohu lake and human gut microbiome calculated by L-ARRAP were displayed in Fig. 5e and f. These results indicated that L-ARRAP could be used to predict ARGs, MGEs, and HBPs in samples from different environment niches and evaluate their ARR.

### Antibiotic resistance risk assessment methods based on long-read sequencing data

The accuracy of the L-ARRAP in identifying ARGs, MGEs, and HBPs and in calculating relative abundance was validated by analysis of environmental samples. Next, we evaluated its risk assessment for antimicrobial resistance. Due to the lack of universal ARG risk assessment methods, we modified MetaCompare for long-reads data and compared its performance with L-ARRAP.

#### Long-read based antibiotic resistome risk assessment pipeline

The abundance analysis of ARG, MGE, HBP, ARG-MGE, ARG-HBP, and ARG-MGE-HBP in samples of before and after disinfection groups showed that there were significant differences before and after disinfection, which also indicated that it was reasonable for ARRI to use these abundances to calculate the risk score.

**Figure 6:**
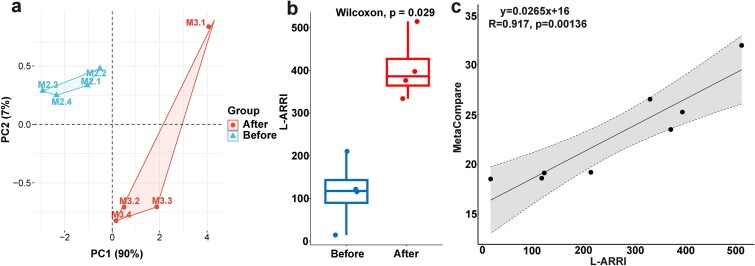
L-ARRI can well reveal the potential risk of ARGs. (a) Principal component analysis showed that samples from before and after disinfection groups could be distinguished according to these abundances of risk assessment model from L-ARRAP. (b) Significant difference of antibiotic resistance risk scores was existed in before and after disinfection groups. (c) Risk scores of L-ARRI were significantly positively associated with results from Rolbiecki D *et al.*

To further evaluate risk score rationality of L-ARRI. First, the samples from before and after disinfection can be separated by these abundances from L-ARRAP (Fig. 6a). Second, the risk scores were significantly higher in postdisinfection HWW samples than in predisinfection samples (Wilcoxon test, *P* = 0.029, Fig. 6b). Furthermore, the risk scores in HWW samples calculated by L-ARRAP and calculated with the method of MetaCompare by Rolbiecki D *et al.* show a significant positive correlation (R^2^ = 0.896, *P <* 0.01, Fig. 6c) [13].

These results showed that the L-ARRI effectively differentiated between samples in before and after disinfection groups, exhibiting a significant positive correlation with abundances of resistome, indicating that L-ARRI accurately reflects the ARR in samples of before and after disinfection groups, thereby validating the rationality of its risk assessment model.

#### L-MetaCompare

**Figure 7:**
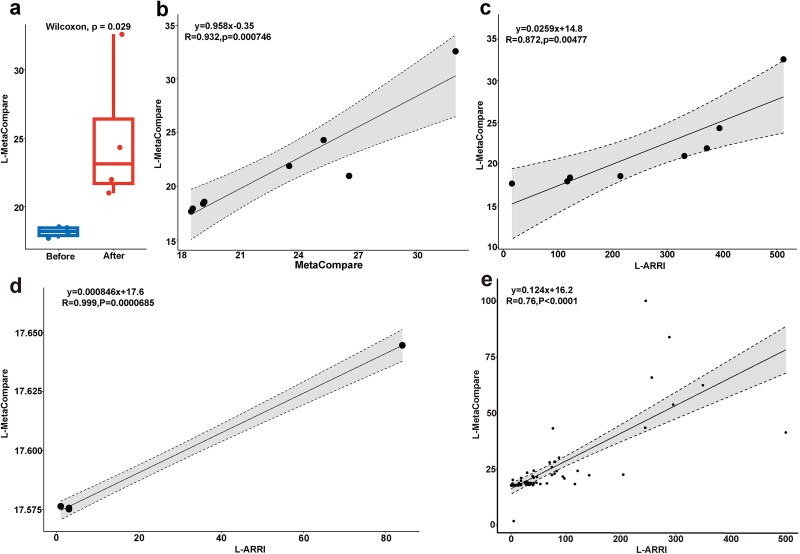
L-MetaCompare also revels well to ARGs risk in samples. (a) There was significant difference in the prediction results of ARGs risk for samples of before and after disinfection groups by L-MetaCompare. (b) The antibiotic resistance risk scores calculated by L-MetaCompare were significantly positively correlated with that calculated by Rolbiecki D *et al.*, which further verified the accuracy of the ARG, MGE, and HBPs identification methods of L-ARRAP. The RS predicted by L-MetaCompare was highly positively associated with the L-ARRI from L-ARRAP in samples of (c) HWW (d) Chaohu lake and (e) human gut microbiome (without H45 sample).

L-MetaCompare was constructed by our lab based on the concept of MetaCompare to compare it with L-ARRAP (Supplementary materials). To evaluate the accuracy of L-MetaCompare, we analyzed HWW samples. The results showed that the ARG risk score (RS) was significantly higher in postdisinfection samples than in predisinfection samples (Wilcoxon test, *P* = 0.029, Fig. 7a). Additionally, the RS calculated by L-MetaCompare was significantly positively correlated with that calculated by Rolbiecki D *et al.* (R^2^ = 0.87, *P* < .01, Fig. 7b). In addition, the correlations of ARG abundance (R^2^ = 0.84, *P* < 0.01, Fig. S3a), MGE abundance (R^2^ = 0.73, *P* < 0.01, Fig. S3b), HBP abundance (R^2^ = 0.55, *P* < 0.05, Fig. S3c), ARG-MGE abundance (R^2^ = 0.96, *P* < .01, Fig. S3d), ARG-HBP abundance (R^2^ = 0.82, *P* < 0.01, Fig. S3e), and ARG-MGE-HBP abundance (R^2^ = 0.96, *P* < .01, Fig. S3f) calculated by L-ARRAP and L-MetaCompare were also analyzed. The findings indicated a positive correlation between RS from L-MetaCompare and RS from Rolbiecki D *et al.*, as well as a notable positive correlation with the six types of abundance value from L-ARRAP.

In particular, results revealed a significant positive correlation between L-ARRI and L-MetaCompare RS in HWW samples (R^2^ = 0.76, *P* < 0.01, Fig. 7c) and Chaohu lake (R^2^ = 0.99, *P* < 0.01, Fig. 7d). When assessing 84 gut microbiota samples with L-MetaCompare, H45 sample's Q(ARG) > 0.01 led to an incorrect ARG risk score of 1.69. L-ARRAP, however, accurately assessed the ARG risk coefficient (Fig. 5f). After excluding this outlier, L-MetaCompare and L-ARRI results for the remaining 83 samples remained significantly correlated (R^2^ = 0.58, *P* < 0.001, Fig. 7e).

Additionally, the L-ARRI calculated by L-ARRAP correlated significantly with L-MetaCompare's Q(ARG) (R^2^ = 0.89, *P* < .01, Fig. S4a), Q(ARG_MGE) (R^2^ = 0.75, *P* < 0.01, Fig. S4b), and Q(ARG_MGE_PATH) (R^2^ = 0.80, *P* < 0.01, Fig. S4c). These results strongly validate the reliability and accuracy of L-ARRAP and L-MetaCompare in assessing ARG risks.

## Discussion

The L-ARRAP was proposed by our lab to unify and rapidly assess the risk of ARGs from Nanopore and PacBio data. In this study, L-ARRAP not only demonstrated excellent performance in the environmental samples of Chaohu lake and HWW, showing its potential application in the detection of ARG in the ecological environment. Furthermore, the results of L-ARRAP show that there are significant differences in the ARGs risk scores among different intestinal samples, demonstrating its potential application in the detection of intestinal microbiota ARG risk in clinical context. L-MetaCompare and L-ARRAP employ different methods to quantify ARGs. L-ARRAP calculates the absolute abundance of ARGs, whereas L-MetaCalculate estimates the proportion of ARG-containing reads within the total reads. Despite this methodological difference, the risk scores generated by both tools showed a strong positive correlation. Furthermore, these scores were highly correlated with the key parameters (abundance and Q-value) used to construct their respective risk prediction frameworks, confirming that both can effectively reflect the risk index of ARG.

While L-ARRAP represents significant progress in ARG risk assessment for long-read datasets, it has several limitations. First, although sequencing technologies such as Nanopore and PacBio are producing increasingly long reads, this very advancement may diminish the significance of the Q(ARG), Q(ARG_MGE), and Q(ARG_MGE_HBP) abundance metrics in L-MetaCompare. This effect, confirmed by Rolbiecki D *et al.* [13] in assembled contigs, undermines the accuracy of resistance risk assessment. Additionally, many studies estimate ARG abundance by normalizing the number of ARG-containing reads to dataset size (Gb) [13, 17, 41]. However, as read length increases, this approach underestimates actual abundance, leading to potential biases.

To address this, L-ARRAP expresses ARG and MGE abundance as the equivalent full-length gene copies per Gb, mitigating read length effects. Nevertheless, since the mobility potential of an ARG is assessed based on its co-localization with MGEs within the same read, L-ARRAP's risk assessment remains influenced by read length. Previous studies suggest that MGEs in close proximity to ARGs are more likely to capture them during the process of transfer [15, 29]. Thus, migration potential is typically assessed by considering MGEs within 5–10 kb upstream or downstream of ARGs [14, 15, 28, 42]. In future versions, we will refine this approach by defining a specific ARG-MGE distance threshold to reduce annotation bias in long-read datasets.

Second, we simulated Minimap2 performance across error rates (1%, 5%, 10%, 15%, and 20%) and found higher rates significantly impair it (Fig. S6). Notably, to ensure result reliability, the dataset’s error rate should not exceed 10%. However, ongoing reductions in error rates from improved Nanopore and PacBio sequencing will enhance the L-ARRAP's accuracy in identifying critical sample components and risk assessment. Third, the increasing evidence suggesting that epigenetics plays a key role in the development of bacterial antibiotic resistance, but it is difficult to incorporate it into the formula when quantifying the risk of ARG [43]. In the subsequent update of L-ARRAP, we will further consider the impact of epigenetics on ARG risk. Finally, L-ARRAP provides few databases and optional tools. In the future, we will expand the database used by L-ARRAP, including HBP database, and provide more tools such as DeepARG [44] to meet the needs of different users.

## Conclusion

With the development of long sequencing technology and its wider application in microbiome research, especially its advantages over Illumina data for ARGs genetic background, there is a lack of ARG risk assessment methods for long-reads. Therefore, this study proposes an ARR assessment pipeline for the first time based on long-reads: L-ARRAP. The results showed that it could accurately identify ARGs, MGEs, and HBPs in samples from different environmental habitats. The risk scores calculated by L-ARRAP were not only significantly different between HWW samples of before and after disinfection groups, but also significantly and positively correlated with RS calculated by Rolbiecki D *et al.*, which indicated that it could accurately respond to the changes in the risk of ARGs in samples of before and after disinfection groups. In addition, we compared the risks of ARGs from the samples of human feces and Chaohu lake that calculated by L-ARRAP and the RS calculated by the MetaCompare's modified method (L-MetaCompare), which also showed a significant positive correlation. Overall, our proposed L-ARRAP is important for the detection, prevention, and management of ARGs in the environmental niches.

**Key Points**

- L-ARRAP was the first pipeline for evaluating ARG risk in long read metagenomic data.
- The L-ARRAP can accurately predict key pathogens, mobile genetic elements, and ARGs from different habitats, and calculate risk scores of antibiotic resistome.
- L-ARRAP can accurately distinguish the ARG risk between pre and post disinfection hospital wastewater samples. Comparing the L-ARRAP method with MetaCompare's modified method (L-MetaCompare), the risk score results showed a high degree of consistency.
- The ARG risk assessment method of L-ARRAP can be applied to long read metagenomic data of various environmental niches.

## Supplementary Material

Supplement-final_bbaf535

Supplementary_Table_bbaf535

## Data Availability

All L-ARRAP code is available on Github (https://github.com/liyonxin/L-ARRI). The Chaohu lake dataset generated and analyzed in this study is available from the corresponding author upon reasonable request. Login codes for real datasets from other studies used in this study were downloaded from the NCBI sequence archive, including PRJNA999488 of HWW, and PRJNA929328 of human gut microbiome.
