# Supplementary material for: Quantifying antibiotic resistome risks across environmental niches: the L-ARRAP for long-read metagenomic profiling
Source: Brief Bioinform. 2025 Oct 9;26(5):bbaf535. doi: 10.1093/bib/bbaf535 (PMC12510455; doi:10.1093/bib/bbaf535)
Supplement: Supplement-final_bbaf535 [file supplement-final_bbaf535.docx]

**Supplementary Materials**

**Quantifying Antibiotic Resistome Risks Across Environmental Niches: The L-ARRAP for Long-Read Metagenomic Profiling**

Yongxin Li^1, 2, †^, Yue Gao^1, 2, †^, Xiaohui Liu^6, †^, Yujie Mao^4^, Mingchao Wang^5, 6^, Yunyi Qin^1, 2^, Caili Zhang^1, 2^, Qingru Chen^1, 2^, Kang Ning^7, *^, Zhi Wang^4, *^, Maozhen Han^1, 2, *^

^1^ School of Life Sciences, Anhui Medical University, Hefei 230032, China

^2^ Microbial medicinal resources development research team, Anhui Provincial Institute of Translational Medicine

^3^ Key Laboratory of Marine Environment and Ecology, Ministry of Education and College of Environmental Science and Engineering, Ocean University of China, Qingdao 266100, China

^4^ Key Laboratory for Environment and Disaster Monitoring and Evaluation of Hubei, Innovation Academy for Precision Measurement Science and Technology, Chinese Academy of Sciences, Wuhan 430077, China

^5^ Qingdao University of Science and Technology, Qingdao 266000, China

^6^ Single-Cell Center, CAS Key Laboratory of Biofuels and Shandong Key Laboratory of Energy Genetics, Qingdao Institute of BioEnergy and Bioprocess Technology, Chinese Academy of Sciences, Qingdao 266100, China

^7^ Key Laboratory of Molecular Biophysics of the Ministry of Education, Hubei Key Laboratory of Bioinformatics and Molecular-Imaging, Department of Bioinformatics and Systems Biology, College of Life Science and Technology, Huazhong University of Science and Technology, Wuhan, China.

^†^ These authors contributed equally to this work.

^*^ Corresponding author E-mail: hanmz@ahmu.edu.cn; zwang@apm.ac.cn; ningkang@hust.edu.cn

**2.Materials and methods**

**2.1 Generation of long sequencing reads for Chaohu water samples**

Four water samples were collected by us in July 2022 from Chaohu Lake (**[Fig.S1](https://www.ncbi.nlm.nih.gov/pmc/articles/PMC11232279/#sup2)**). A total of 2 L water at a depth of 0.5 m was collected at each site for water samples using a cylinder sampler, and filter the collected water through a 0.22 μm nitrocellulose membrane to collect the solids. The membrane samples were immediately stored in a portable cooler with ice bags, transported to the laboratory, and stored at -80 ℃. Four water samples were collected randomly by us in July 2022 from Chaohu Lake (Fig.S1). Then, the water samples were sequenced on Nanopore sequencing platform. Specifically, total DNA was extracted and electrophorezed using the DNeasy PowerSoil Kit (Qiagen, Germany) and recovered using the Monarch^®^ DNA Gel Extraction Kit (NEB Inc., USA). AMPure XP beads (Beckman Coulter) were used to purify the recovered DNA. DNA samples with sufficient purity (A260/230 ≈ 1.8, A260/280 ≈ 2.0) were purified to end-pairing and DA-tailing by NEBNext Ultra II End Repair/dA Trailing Module. Finally, libraries were loaded onto flow cell versions FLO-MIN106 R9.4 SpotON and sequenced for 16 h. Details of all metagenomic samples are shown in **Supplementary Table S1** and the bioinformatic analysis of long-sequencing reads was provided in Supplementary Material

**2.2 ARGs and MGEs identification**

To identify ARGs, the best cutoff values of identify/coverage were optimized. A combined dataset of 125,190 non-ARGs bacterial genes randomly downloaded from the KEGG database and 14,210 ARGs genes from the SARG database was constructed to verify the ARGs annotation precision of L-ARRAP for different cutoff values. Only hit with identical subtype were counted as valid ARGs identification. As shown in **Fig.1b**, ARGs identification with an identity cut-off of 75% showed that precision greater than 95% and the percentage of ARGs identified greater than 99%, which indicates effective filtering of false-positive hits at this cut-off. A further increase in threshold of identity would have little effect on precision, but would significantly increase false negatives for ARG identification (**Fig.s2**). For example, when the threshold of identity is raised to 0.8, the percentage of identified ARGs drops below 90%. Consequently, L-ARRAP adopted an identity cut-off of 75% and coverage cut-off of 90% for ARGs identification. L-ARRAP calls the Minimap2 (preset map-ont, map-pb or map-hifi for different reads) to align the reads with the nucleotide sequences of the SARG database v2, and only the ARG hits with the highest mapping quality were kept whenever hits overlapped with other ARG hits by 80% in length to avoid multiple hits on the same gene segment.

Due to the fact that MGEs are able to capture ARGs from chromosomes and transfer them horizontally to other bacteria via plasmids or phages, MGEs should be paid attention to when assessing the risk of antibiotics. In this study, MGEs were identified using LAST tool (v2.27.1) to align the reads with the protein database of mobileOG-db (Version: Beatrix 1.6 v1). There, each of MGEs were scanned by researchers of MobileOG-db to determine whether the gene name might encode a protein that performed one of the target functions: the integration/excision, replication/recombination/repair, stability/defense, or transfer of bacterial mobile genetic elements and phages as well as the associated transcriptional regulators of these processes. The identity cut-off of 75% and coverage cut-off of 90% is adopted by L-ARRAP, and only the MGE hits of the highest mapping quality were kept whenever hits overlapped with other MGE hits by 80% in length to avoid multiple hits on the same gene segment.

**3 Result**

**3.1 L-MetaCompare**

MetaCompare is an antibiotic resistance risk prediction pipeline developed based on short-read sequencing metagenomic data, with the standard input data being contigs assembled from short reads. The MetaCompare pipeline involves three quantities when calculating the antibiotic resistance risk score, namely, Q(ARG), Q(ARG_MGE), Q(ARG_MGE_PATH) represent, respectively: the percentage of contigs containing ARGs, the percentage of contigs annotated with ARG and MGE, the percentage of contigs annotated with pathogen genomes as well as ARGs and MGEs. If the long-read sequencing data is assembled into contigs, it will be very long, and the probability of including ARGs and MGE in contigs will be greatly increased. Therefore, calculating the proportion of the number of contigs containing ARGs to the total contigs does not correctly reflect the abundance of ARGs in the sample. In addition, Rolbiecki D et al. have assembled the long reads and found that the Q (ARG) of the assembled contigs has been greater than the 0.01 maximum value preset by MetaCompare.

Therefore, L-MetaCompare was constructed by us, an antibiotic resistance risk assessment method for long reads, based on the MetaCompare. There, contigs were replaced with unassembled long-reads to calculate Q(ARG), Q(ARG_MGE), Q(ARG_MGE_PATH) that were detected by L-ARRAP. In MetaCompare, the maximum 'h' value is 0.01, determined by cutting pathogen genomes based on contig lengths from Illumina data. However, the distribution of read lengths differs across samples, with longer reads leading to larger theoretical 'h' values in L-MetaCompare, making it difficult to set a fixed 'h'. Thus, L-MetaCompare retains the maximum 'h' value of 0.01 from MetaCompare. Note that in this study, one human gut microbiome sample had a Q(ARG) greater than 0.01, leading to inaccurate results. Therefore, when using L-MetaCompare, ensure that the Q(ARG) value is less than 0.01.


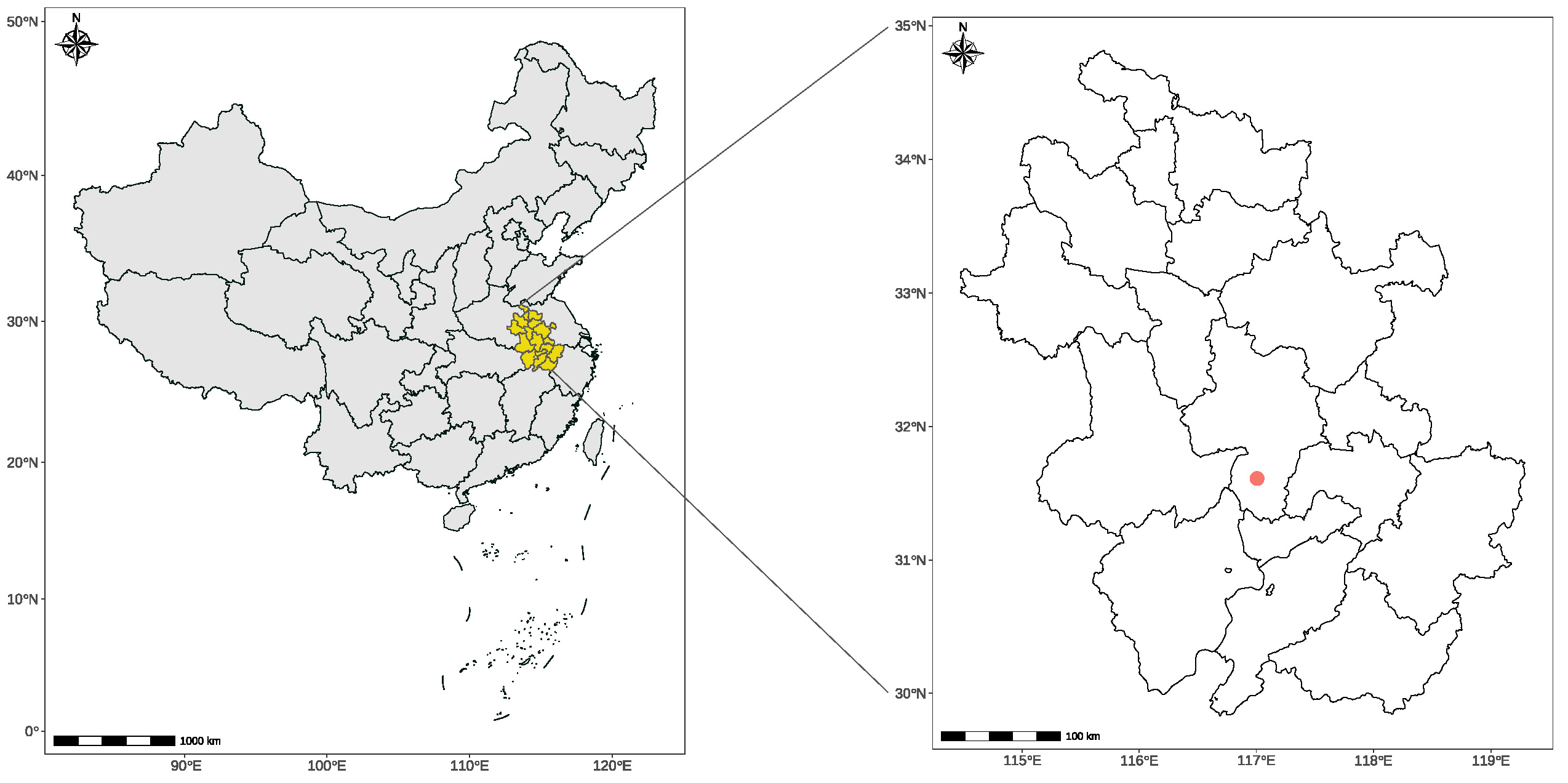


**Fig S1.** The location of Chaohu Lake was indicated with the red dot on the map.


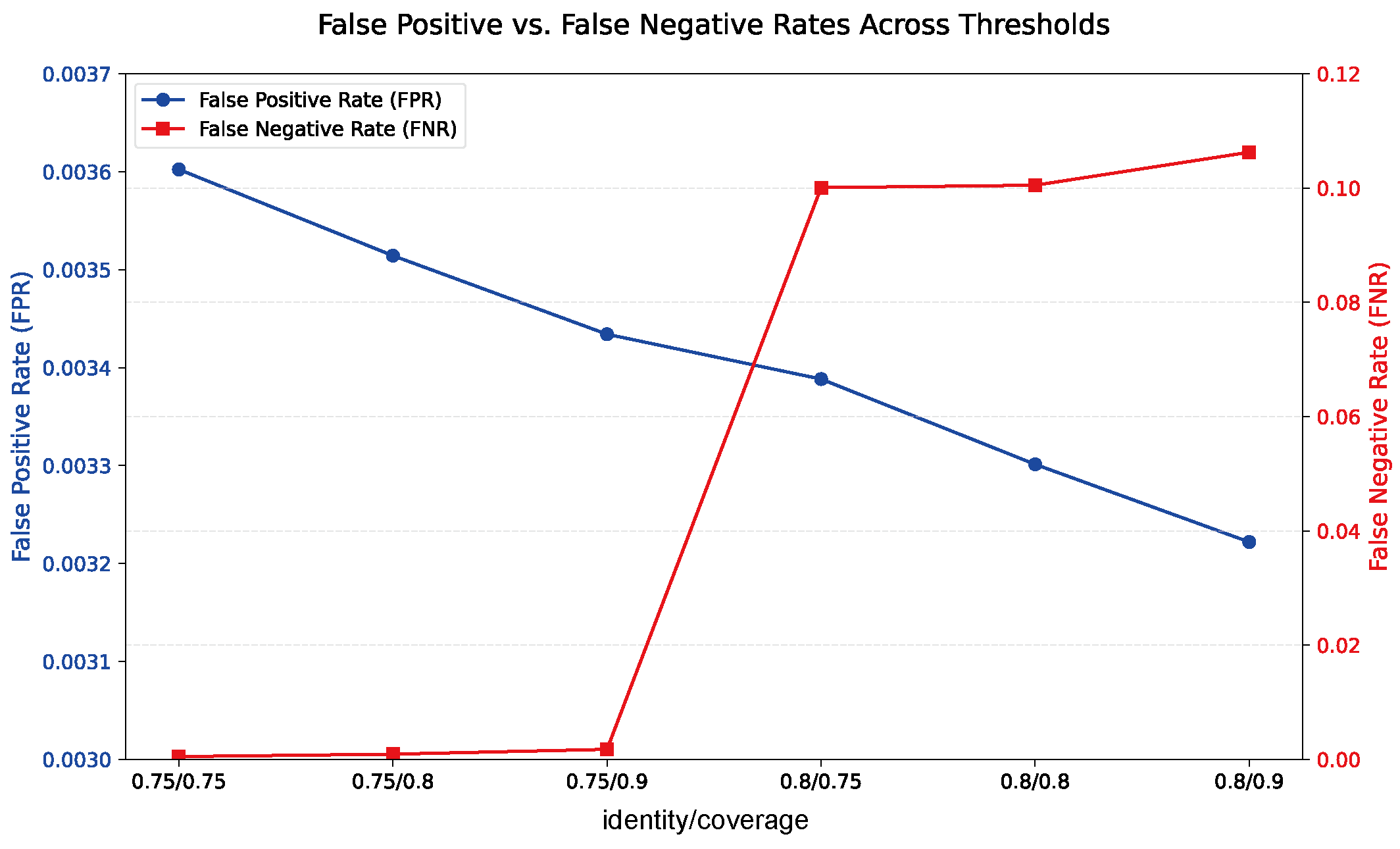


**Fig S2.** The false positive rate and false negative rate of ARGs identified with the different identity/coverage cut-off value by L-ARRAP.


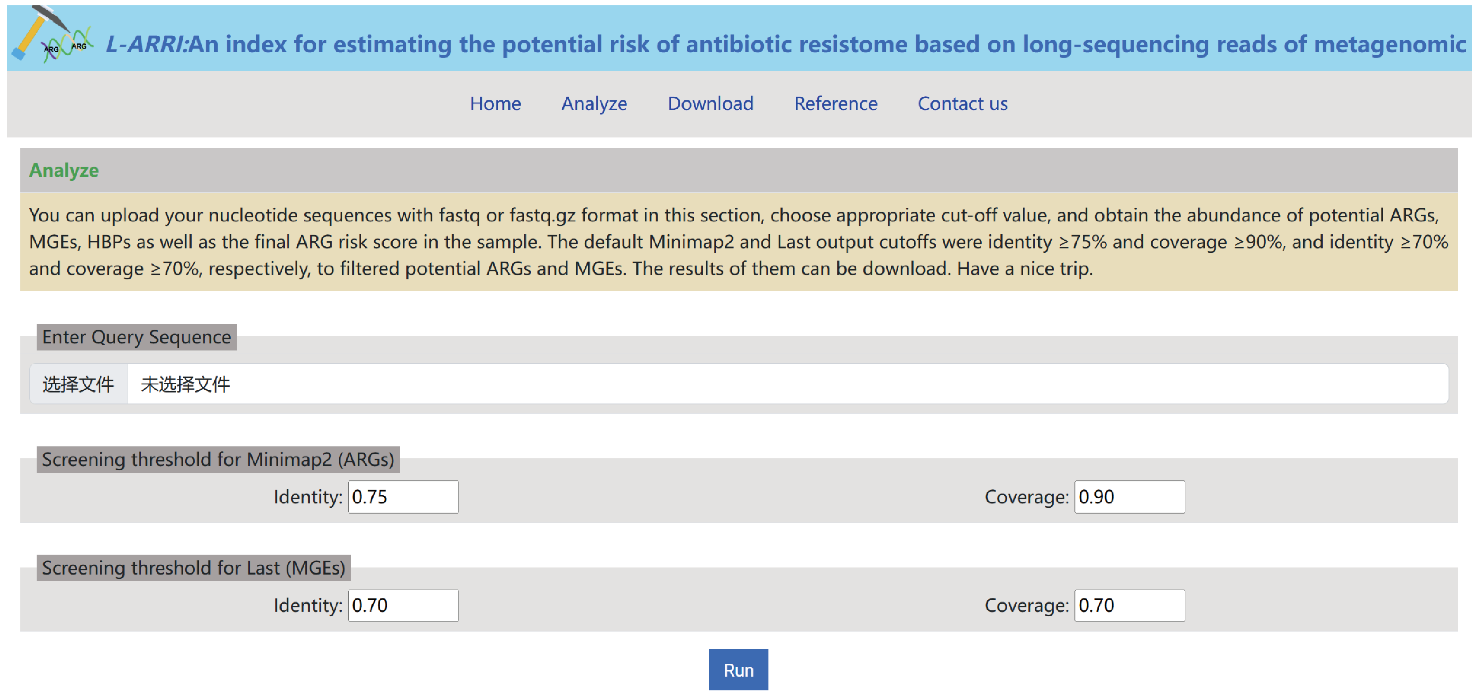


**Fig.S3** **The website of L-ARRAP for estimating the potential risk of antibiotic resistome based on long-sequencing reads of metagenomic data.**


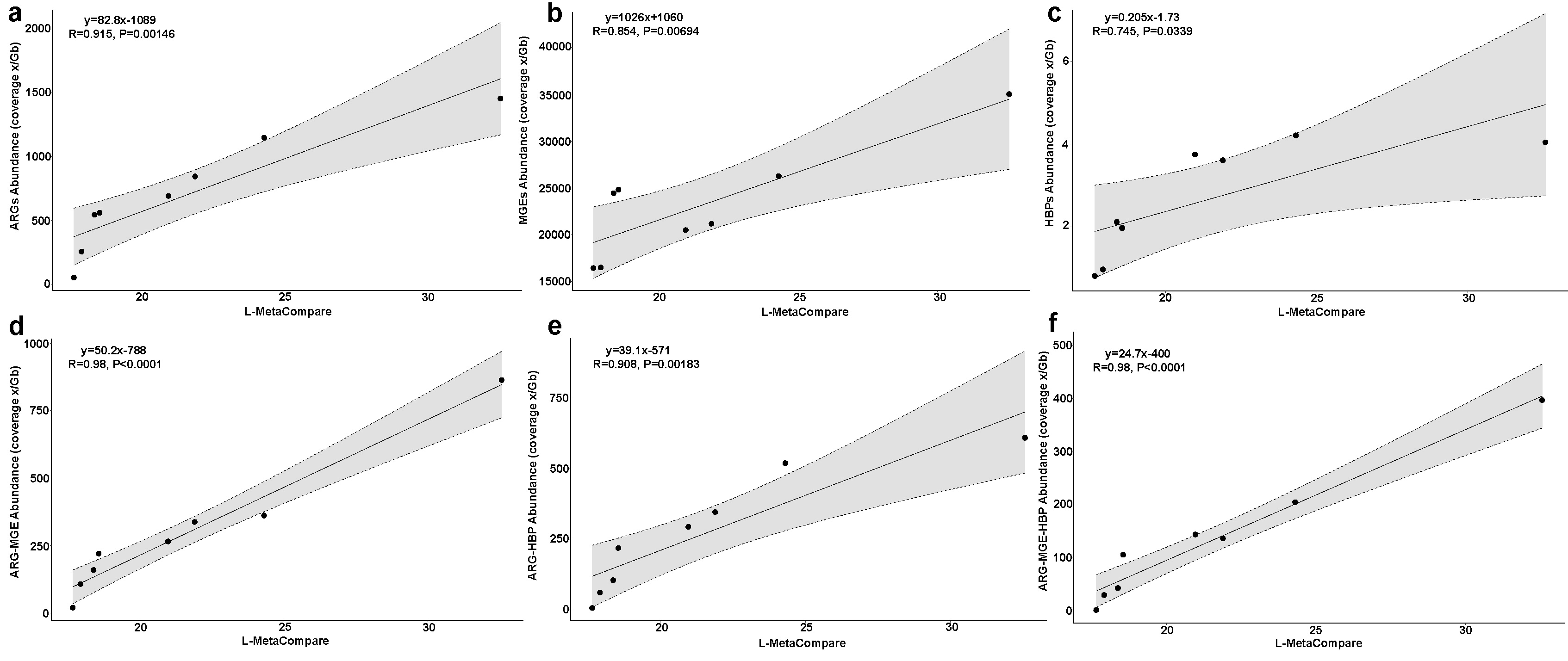


**Fig S4.** The risk scores calculated by L-MetaCompare for HWW samples were significantly positively correlated with the abundances of (a) ARGs, (b) MGEs, (c) HBPs, (d) ARG-MGE, (e) ARG-HBP and (f) ARG-MGE-HBP that comprise the L-ARRI risk index.


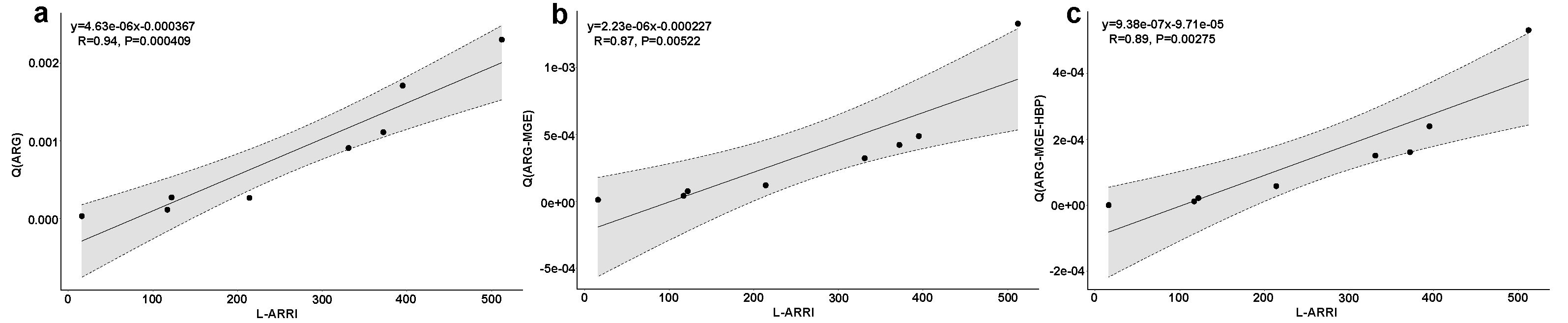


**Fig S5.** A strongly significant correlation existed between the L-ARRI values and (a) Q(ARG), (b) Q(ARG_MGE) and (c) Q(ARG_MGE_PATH).


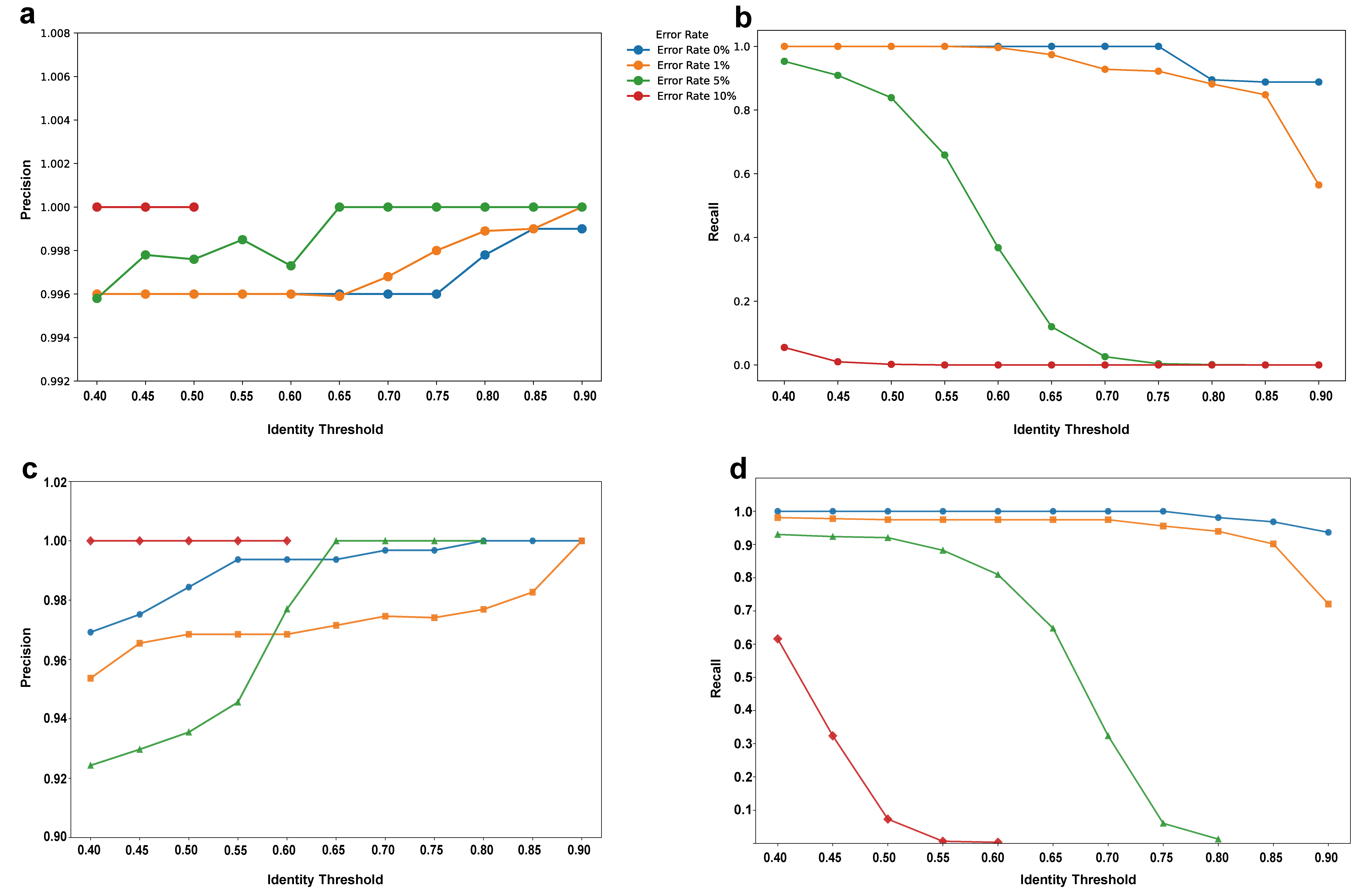


**Fig.S6** Effects of different error rates on the precision and recall of Minimap2 in ARG identification. A combined dataset of 1000 ARGs randomly selected from the SARG database and 1000 non-ARGs randomly downloaded from the KEGG database was constructed to explore the effect of sequencing error rate on Minimap2. We simulated the performance of Minimap2 at different error rates (1%, 5%, 10%, 15%, and 20%), and found that higher error rates had a significant impact on Minimap2 (**Fig.S6a,b**). When the error rate exceeded 15%, ARGs could hardly be identified, therefore the lines of 20% was not shown in the figure. To further explore the effect of error rate and threshold selection on the performance of minimap2 in different datasets, we randomly downloaded 50 bacterial genomes and simulated different error rates. It also show that high error rates significantly affect minimap2's performance (**Fig.S6c,d**). Moreover, at higher error rates, increasing the identity threshold slightly improves precision but significantly reduces recall. Therefore, when the error rate is high, the threshold can be appropriately lowered to increase the recall rate.
